# Sarcoptic mange in a guanaco (*Lama guanicoe*) of northwestern Argentina: Clinical, histopathological and molecular studies

**DOI:** 10.1016/j.ijppaw.2025.101062

**Published:** 2025-03-26

**Authors:** Fabiana E. Sosa, Melina Anello, Hebe del Valle Ferreyra, Diego M. Medina, Juan F. Micheloud, Hugo M. Borsetti, Nancy Hernández, Mónica Florin-Christensen, Sandra R. Romero

**Affiliations:** a Instituto de Investigación y Desarrollo Tecnológico para la Agricultura Familiar – Región NOA (IPAF NOA), Instituto Nacional de Tecnología Agropecuaria (INTA), Posta de Hornillos (4624), Argentina; b Consejo Nacional de Investigaciones Científicas y Técnicas (CONICET), Buenos Aires, C1425FQB, Argentina; c Grupo de Investigaciones en Ecología y Fisiología de Fauna Silvestre, Instituto de Investigaciones en Biodiversidad y Medio Ambiente, CCT-Patagonia Norte (AUSMA- GIEFAS-INIBIOMA-CONICET), San Martin de los Andes, 8370, Argentina; d Instituto Multidisciplinario de Biología Celular, CONICET-UNLP-CIC, La Plata, B1906APO, Argentina; e Administracion de Parques Nacionales, Buenos Aires, C1033AAE, Argentina; f Área de Sanidad Animal, Instituto de Investigación Animal del Chaco Semiárido (IIACS), Centro de Investigaciones Agropecuarias (CIAP), INTA, Cerrillos, 4403, Argentina; g Instituto de Estudios Celulares, Genéticos y Moleculares (ICeGeM), Universidad Nacional de Jujuy (UNJU), San Salvador de Jujuy, 4600, Argentina; h Instituto de Ecorregiones Andinas (INECOA), CONICET-UNJU, San Salvador de Jujuy, 4600, Argentina; i Instituto de Patobiología Veterinaria (IP-IPVET), Centro de Investigaciones en Ciencias Veterinarias y Agronómicas, INTA-CONICET, Hurlingham, 1686, Argentina

**Keywords:** Guanaco, Sarcoptic mange, Mites, Genotyping, Conservation

## Abstract

Sarcoptic mange, caused by the mite *Sarcoptes scabiei*, is a highly contagious and potentially fatal skin disease that affects a wide range of mammals, including South American Camelids (SAC). Although the presence of mange has been described in vicuñas and llamas in northwestern Argentina, there are no previous records documenting its presence in guanacos (*Lama guanicoe*) of this region. We here describe a case of *S. scabiei* in a free-ranging guanaco in the Department of Tilcara, Jujuy Province. The animal presented alopecic, erythematous and hyperkeratotic lesions with abundant crusts in the ventral region of the body and limbs. Histopathology revealed periadnexal dermatitis with inflammatory infiltrate, severe hyperemia, orthokeratotic and parakeratotic epidermal hyperplasia, and stratum corneum thickening. The etiological agent was identified as *S. scabiei* by microscopic examination of adult mites. This result was confirmed by sequencing of a cytochrome oxidase subunit I gene fragment, that showed 100 % identity with sequences of isolates from SAC and other mammals. Genotyping of three mites isolated from different parts of the guanaco's body using a set of ten microsatellite markers indicated the infection with a single genetic variant that showed a similar profile to those found in *S. scabiei* isolates from vicuñas and llamas of the same region. However, genetic differences with guanaco isolates from midwestern Argentina were observed. This work presents the first record of sarcoptic mange in a guanaco in Jujuy Province and in the northwestern region of Argentina, as well as the molecular characterization of the etiological agent. The case highlights an uncertain scenario regarding the health situation of the guanaco population in this region, which is small, fragmented, and locally categorized as endangered. Epidemiological surveillance programs for guanacos and further research on the impact of sarcoptic mange on the conservation of this species are needed.

## Highlights

• This is the first report of sarcoptic mange in guanacos of the Argentine Northwest.
• Clinical and histopathological signs were as those found in free-roaming vicuñas.
• Mites were identified as *Sarcoptes scabiei* by morphology and cox-1 sequencing.
• Mites were genetically close to isolates from other camelids in the same region.
• Genetic differences with guanaco isolates from midwestern Argentina were found.

## Graphical abstract

**Figure:**
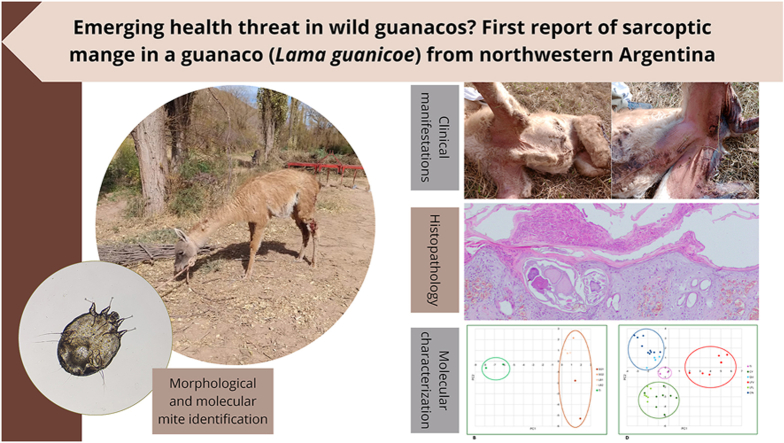

## 1 Introduction

The guanaco (*Lama guanicoe*) is a wild representative of the South American camelids (SAC), a group also comprising the wild vicuña (*Vicugna vicugna*), and the domesticated llama (*Lama glama*) and alpaca (*Vicugna pacos*). It is distributed across a wide range of environments in Peru, Bolivia, Chile, Paraguay, and Argentina (Franklin, 1982; Carmanchahi et al., 2022).

The guanaco is globally classified as a species of "Least Concern" by the International Union for Conservation of Nature (Baldi et al., 2016). In Argentina, however, the situation varies significantly by region. Indeed, guanaco populations are generally composed of numerous individuals in Patagonia, but they are small and fragmented in the central and northern areas of the country, where the species is classified as locally endangered (Carmanchahi et al., 2019, 2022). The latest census carried out in 2006 reported less than 1000 specimens in Jujuy, a province in the northwestern tip of Argentina (Baigún et al., 2008). Guanacos are affected by various environmental issues, such as habitat loss, livestock introduction, desertification due to overgrazing, hunting, and climate change (Fernández and Stecher, 2022). Furthermore, pathogens might pose an additional threat to this species. Although the information on the sanitary situation of guanacos is scarce, cases of fasciolosis and clostridiosis have been reported in Argentina (Uzal et al., 2000; Issia et al., 2009).

Sarcoptic mange, a highly transmissible ectoparasitic disease produced by *Sarcoptes scabiei* mites, affects numerous mammalian species including wildlife, domestic animals, and humans. At first, the disease is manifested by non-pruritic erythematous plates. These develop into seborrheic dermatitis and a hypersensitivity response accompanied by intense pruritus. In critical cases, it can progress into severe crustal dermatitis with chronic inflammation extending to large body areas. This can lead to difficulty in walking and, consequently, foraging and/or escaping predators and other aggressors, as well as weakness, dehydration, emaciation, and death (Pence and Ueckermann, 2002; Martin et al., 2018).

Sarcoptic mange has been reported in free-roaming guanacos of Chile and Argentina as well as in captive guanacos in Greece (Papadopoulos and Fthenakis, 2012; Montecino-Latorre et al., 2020; Ferreyra et al., 2022). Reports in other SAC within their natural distribution area involve vicuñas from Bolivia, Peru and Argentina, and llamas from Argentina (Beltrán-Saavedra et al., 2011; Arzamendia et al., 2012; Gomez-Puerta et al., 2021; Serrano-Martínez et al., 2024; Ferreyra et al., 2022; Sosa et al., 2022, 2025). Sarcoptic mange hampers the well-being of SAC and has an economic impact since it affects the quality and quantity of fiber. It can also have devastating effects in camelid populations as was the case of an outbreak at San Guillermo National Park and its surroundings, in the central-western region of Argentina, where massive mortality of vicuñas and guanacos associated with this infestation led to the local ecological extinction of these species (Ferreyra et al., 2022; Monk et al., 2022).

Systematic studies on mange in SAC populations are crucial for designing and implementing appropriate management strategies for wild and domestic species. The present study constitutes the first sarcoptic mange report in a guanaco of the province of Jujuy, in Argentina Northwest. The clinical and histopathological manifestations of the disease are described, as well as the molecular genotyping of the infecting *S. scabiei* isolate.

## 2 Materials and methods

### 2.1 Sample collection

**Fig. 1:**
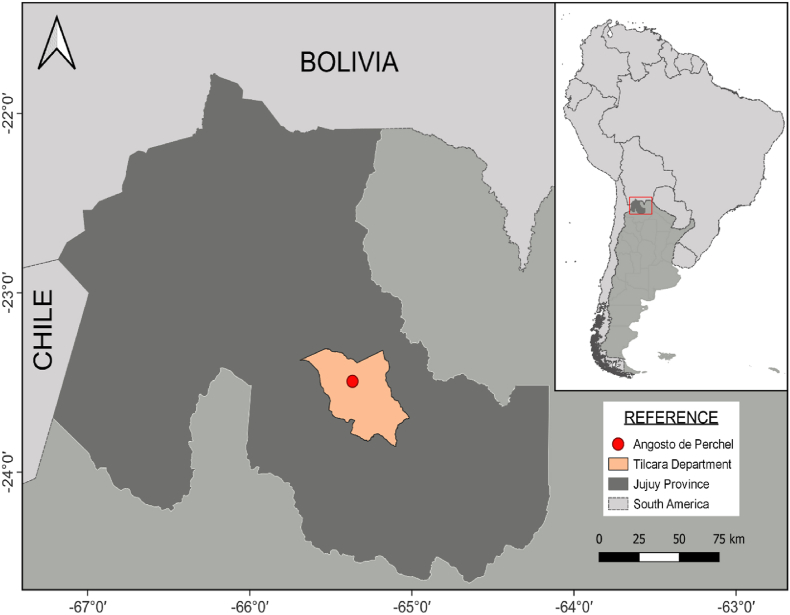
Geographic reference of the location where a guanaco affected by sarcoptic mange was found. Left: Map of Jujuy Province, Argentina, indicating Angosto de Perchel, Tilcara Department (red dot in pink area), in the Quebrada de Humahuaca. Right: Location of the detailed region within South America.

During July 2023, the Center for Native Fauna Care of the Jujuy province, Argentina (CAFAJU), received notice from the firefighters of the town of Tilcara, reporting the rescue of a guanaco that was attacked by dogs in Angosto de Perchel (23°29′37.02’’S 65°21′58.50’’W) (Fig. 1; Fig. S1). The team that immediately responded to the warning, made up of firefighters and CAFAJU personnel, mentioned that the animal was in a poor general condition, with evident weakness and marked difficulty moving. They identified wounds with profuse bleeding located in the posterior region of the thighs, which required immediate veterinary attention. Also, lesions consistent with sarcoptic mange were recorded. The following biomedical samples were collected: (i) skin biopsies from the thorax, abdomen, and left forelimb obtained with disposable biopsy punches and preserved in 10 % buffered formalin, pH 6.9 (Sigma Aldrich, St. Louis, USA), for histopathological studies; and (ii) skin scrapings with and without petroleum jelly from different parts of the body for mite isolation and analysis.

### 2.2 Histopathological studies

Formalin-fixed biopsies were embedded in paraffin and then 3–5 μm sections were obtained using a microtome. Sections were dehydrated and stained with hematoxylin-eosin following standard procedures and observed microscopically (100X).

### 2.3 Morphological and molecular identification of mites

The samples obtained with petroleum jelly were spread on glass slides and observed under light microscopy (400X). Taxonomic identification based on morphological characteristics was carried out according to Arlian and Morgan (2017). DNA was extracted using a commercial kit from a pool of 10 mites from a skin-scraping sample obtained without petroleum jelly (GTI-1 isolate). A *Sarcoptes scabiei*-specific 374 pb fragment of the cytochrome oxidase subunit 1 (*cox-1*) gene was amplified by PCR using the extracted DNA as template, as described by Sosa et al. (2022). Both strands of the PCR product were sequenced at Macrogen (Seoul, Korea) and a consensus sequence was obtained after alignment with BioEdit and deposited in GenBank under accession number PV019091. Highly similar nucleotide sequences were searched in GenBank using BLASTn (https://blast.ncbi.nlm.nih.gov/Blast.cgi) and compared using Clustal Omega (https://www.ebi.ac.uk/jdispatcher/msa/clustalo).

### 2.4 Molecular characterization

DNA from 3 mite individuals (GTI-2, GTI-3, GTI-4 isolates) randomly isolated from different body regions was extracted following the HotSHOT Plus ThermalSHOCK technique (Alasaad et al., 2008), and ten mite microsatellite markers (SARMS 33, 34, 35, 36, 37, 38, 40, 41, 44, and 45) were simultaneously amplified by multiplex PCR as described previously (Rasero et al., 2010; Moroni et al., 2021). PCR products were separated by electrophoresis using SeqStudio Genetic Analyzer and microsatellite fragment sizes and alleles were assigned with GeneMapperTM software.

Microsatellite results were compared with those previously obtained with *S. scabiei* isolates from vicuñas and llamas of Jujuy Province and guanacos of San Juan and La Rioja Provinces (Ferreyra et al., 2022) (Table S1).

A principal component analysis (PCA) was performed via the R package “adgenet” in addition to a Bayesian clustering analysis with the STRUCTURE program (Pritchard et al., 2000). For the latter, a burn-in period of 100,000 followed by the same number of MCMC replications was used. An admixture ancestry model with sampling location as prior information was selected. The number of populations (K) varied from 1 to 7, and 10 iterations for each K were performed. The assignment of the most likely K was made considering the maximum value of ln [Pr (X/K)] and the individual assignment values (q values) (Evanno et al., 2005).

## 3 Results

### 3.1 Clinical findings

**Fig. 2:**
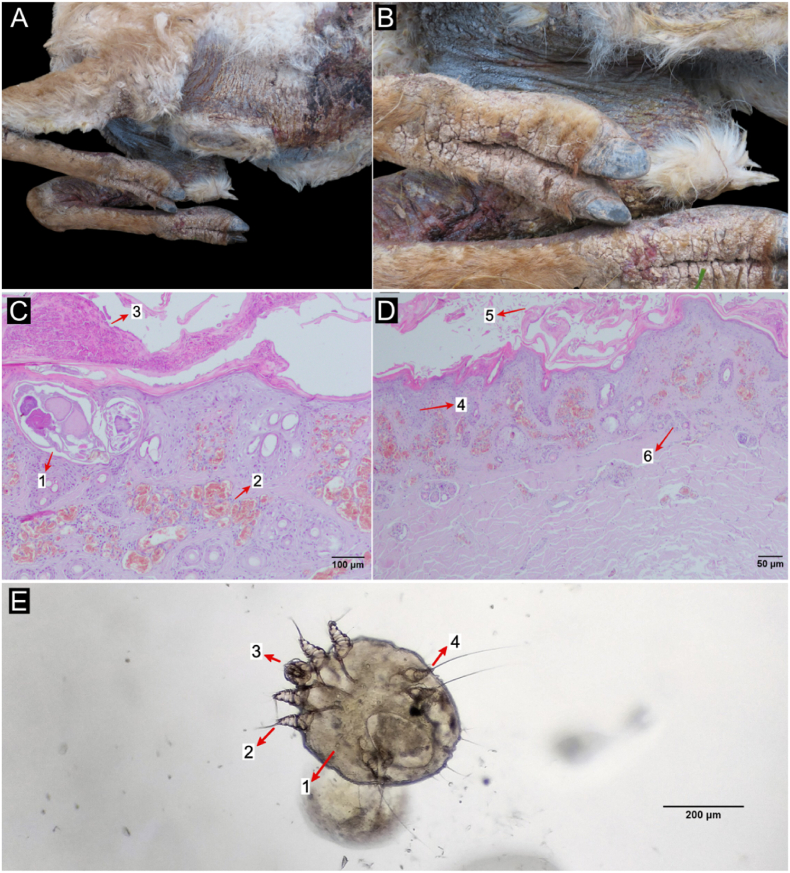
Sarcoptic mange manifestations in a guanaco from Angosto de Perchel. (A,B) Clinical signs: (A) Bleeding scabby lesions, cracks and fiber loss in the ventral region of the animal (chest, armpits, abdomen and forelimbs); (B) Detail of injury in the distal forelimbs, chest and axillary region. The silver coloration in the axillary region and chest is due to the application of medications upon the guanaco's arrival at the wildlife care center. (D,E) Histopathological signs: (1) *Sarcoptes scabiei* mite, (2) severe hyperemia, (3) intraepidermal pustule, (4) epidermal hyperplasia, (5) thickening of the stratum corneum, (6) periadnexal infiltrate. (E) Micrograph (400X) of a *S. scabiei* adult female mite found in a skin scraping: (1) globular idiosome, (2) legs I and II extended over the anterior edge of the idiosome with a tarsus that bears long non-segmented pedicels that end in suction cups, (3) thick gnathostome, (4) legs III and IV ending in long setae corresponding to a female. Note the presence of an egg in the ventral area.

Upon clinical assessment of the guanaco, diffuse, alopecic, and hyperkeratotic lesions, with whitish crusts containing serosanguineous exudate, erythema, deep cracks, and a pronounced tallow odor were observed. The lesions were predominantly located on the limbs and body areas with less fiber coverage, including the lower abdomen, chest, axilla, and groin (Fig. 2A and B). In addition, injuries caused by dog bites with profuse bleeding were observed in different parts of the body, especially in the posterior region of the thighs (Fig. S1). Despite emergency treatment to stabilize the animal and stop the bleeding, the guanaco died a few hours after rescue.

### 3.2 Histopathology

Microscopical examination of histological sections revealed mild to moderate periadnexal dermatitis with a mixed inflammatory infiltrate, severe hyperemia, orthokeratotic and parakeratotic epidermal hyperplasia, and intraepidermal pustules. Mites with *S. scabiei*-consistent morphology were observed at the dermal-epidermal junction. Thickening of the stratum corneum and crusts were also registered (Fig. 2C and D).

### 3.3 Morphological and molecular identification of mites

Skin scrapings contained 0.1–0.4 mm-long mites, corresponding to the stages of larvae, nymphs, and adults. The morphology of the adults was consistent with *S. scabiei*, i.e. they presented a globular idiosome with setae and cuticular spines at the dorsal surface, thick gnathostome, short and stubby legs, and legs I and II extended over the anterior edge of the idiosome with a tarsus carrying long, non-segmented pedicels ending in suction cups, while legs III and IV of females and legs III of males ended in long setae. No isolate eggs were detected in the scrapings, but some fertilized female mites carrying an egg were observed (Fig. 2E).

PCR-amplification of a *S. scabiei*-specific fragment of the *cox-1* gene using GTI-1 DNA as template yielded a band of the expected size (between 300 and 400 bp). The sequenced amplicon (PV019091) showed 100 % identity with partial *cox-1* sequences of *S. scabiei* isolates from vicuña (OL739582, OL739583, OL739584) and llama (PP748549, PP748550) from Jujuy province, koala from China (MF083743.1), dog from China (KJ748528) and human from Saudi Arabian (OK310847) (Fig. S2).

Thus, the identity of the etiological agent was confirmed as *S. scabiei* both by morphological and molecular studies.

### 3.4 Molecular characterization of the guanaco S. scabiei isolate

**Fig. 3:**
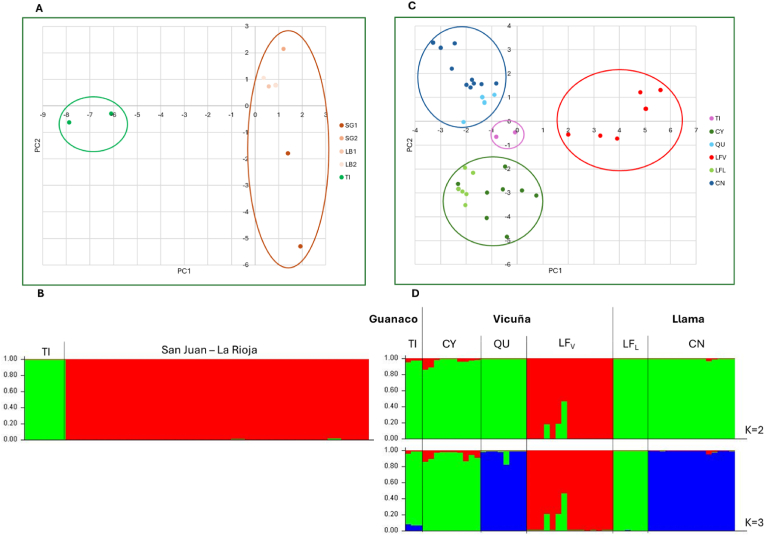
Genetic characterization of *S. scabiei* isolates from a Tilcara guanaco. Genetic profile comparisons obtained with a microsatellite set were carried out by Principal Component Analysis (PCA) (A,C) and Bayesian clustering (B,D). The Tilcara isolates were analyzed together with guanaco mite isolates from other regions of Argentina (A,B) and with llama and vicuña mite isolates from Jujuy (C,D). PCA scatter plots include components 1 *vs*. 2 and each color-coded dot represents a single mite from the corresponding sampling site, as indicated by the reference panel at the right of the plots. The main groups are encircled with different colors. Structure plot for K clusters 2, and for K = 2 and 3 are plotted; each vertical line represents an individual mite. CN: llamas of Cieneguillas; QU: vicuñas from Quera; TI: guanaco from Tilcara (GTI-2, 3, 4); LFL: llamas from Lagunilla del Farallón; CY: vicuñas from Coyaguayma.

Identical alleles for the ten microsatellites were obtained with DNA of the *S. scabiei* Tilcara guanaco isolates (GTI-2, GTI-3 and GTI-4), with the exception of a missing allele in SARM 33 for GTI-2. When comparing these genotypes with those of mites isolated from guanaco samples from San Juan and La Rioja Provinces (n = 22), both PCA and Bayesian cluster analysis showed two separate groups (Fig. 3A and B). On the other hand, genetic similarities were found between the Tilcara guanaco isolates and those isolated from llamas (n = 21) and vicuñas (n = 33) from other localities of Jujuy province. According to the PCA results, the first two components account for 8.78 % and 4.56 % of the variation, respectively. Three groups were differentiated for llamas and vicuñas of Jujuy (Fig. 3C): one is formed only by mites from vicuñas of Lagunillas del Farallón (LFV, red circle), while the other two contain isolates from both llamas and vicuñas from different parts of the province (green and blue circles). The Tilcara isolates, which form a fourth group (purple circle) are genetically closer to the two latter shared groups. Structure analysis supported these results (Fig. 3D). For K = 2, LFV mites formed a single cluster while the rest of the mites from Jujuy SAC (guanaco, llamas, and vicuñas from other locations) grouped in a second cluster. For K = 3, which was the most probable number of clusters, the first group consisted of LFV, the second one contained isolates from llamas of Cieneguillas (CN) and vicuñas from Quera (QU), and the third, contained the Tilcara guanaco isolates (TI) together with isolates from llamas of Lagunilla del Farallon (LFL) and vicuñas from Coyaguayma (CY).

## 4 Discussion

This study constitutes the first report of sarcoptic mange affecting guanaco in the northwestern region of Argentina. Mange was detected in only one individual, thus the extent of the disease at the population level remains unknown. However, given the potentially devastating effect of sarcoptic mange on wild camelids, and that the event occurred in a region where the guanaco is endangered, this episode should not go unnoticed.

The guanaco evaluated in this study presented severe diffuse lesions characterized by total or partial alopecia, hyperkeratosis, abundant crusts, erythema, and bleeding, extensively affecting the ventral region. These findings are consistent with descriptions of advanced stages of sarcoptic mange documented in guanacos from the San Guillermo National Park, Argentina (Ferreyra et al., 2022), where 33 % of the observed living guanacos and 85 % of the examined carcasses presented similar lesions. Likewise, Papadopoulos and Fthenakis (2012) described hyperkeratotic lesions and crusts, located in the neck, shoulder, limbs, and thoracic region, together with intense pruritus in two captive guanacos affected by sarcoptic mange.

The clinical characteristics observed in the guanaco of the present study are also comparable to those reported in vicuñas from Argentina and Peru (Sosa et al., 2022; Ferreyra et al., 2022; Serrano-Martínez et al., 2024), as well as to those reported in llamas with parakeratotic mangy lesions from Argentina (Sosa et al., 2025). From a histopathological perspective, results from the guanaco biopsies coincide with those reported for mangy guanacos by Ferreyra et al. (2022), including inflammatory infiltrate, epidermal hyperplasia, hyperemia, and the presence of mites at the dermo-epidermal junction. These results highlight a common pattern in the clinical manifestation of the disease, regardless of camelid species or geographic region.

Morphological features, as well as the sequence of a *cox-1* gene fragment, were used to identify the etiological agent as *S. scabiei*. No polymorphism was observed between the *cox-1* fragment from guanaco and the corresponding sequences of *S. scabiei* isolates from other hosts and origins. While this could be due to the short length of the obtained sequence (348 bp), it is noteworthy that no polymorphism was observed either among longer *cox-1* sequences (∼1000 bp) from llama, guanaco, and vicuña *S. scabie*i isolates from different regions of Argentina in a recent report (Anello et al., 2025). These results indicate that the use of *cox-1* as a molecular marker for *S. scabiei* strain typification might be limited.

Microsatellite markers, on the other hand, allowed the genetic characterization of this isolate. Our results indicate that a single genetic variant infected this guanaco, since the three randomly isolated mites from different parts of the body presented the same patterns. This result is different from the co-infection with different mite genetic variants observed in other sarcoptic mange cases in SAC from Argentina (Anello et al., 2025). Genetic comparison of the Tilcara guanaco isolates with isolates from llamas and vicuñas from Jujuy showed that the mite genetic variant that affected the guanaco is not species-specific, although a host family-based parasite adaptation is possible. Similarly, other studies support the transmission among wild and domestic SAC (Ferreyra et al., 2022). Transmission could take place directly through contact between healthy and sick individuals, or indirectly through surfaces contaminated with mites such as fences or wallows, the latter of which are shared by individuals of the same group, by neighboring groups, and by domestic camelids (Serrano-Martínez et al., 2024; Arzamendia et al., 2012).

Nevertheless, the shared genetic profile among mite isolates from different SAC of Jujuy Province also points to a geographically related transmission that is attributable to the overlap of habitats and/or the close cohabitation of these species in the territory. Cross-transmission of *S. scabiei* between SAC and other species has also been observed before in enclosed conditions (Papadopoulos and Fthenakis, 2012; Pisano et al., 2018) and suspected in the wild (Gomez-Puerta et al., 2024). This situation could facilitate the spread of the disease in the studied region, though further investigations into *S. scabiei* cases in other wild and domestic hosts that share the same territory would help clarify the mite transmission mechanisms.

The existence of multiple genetic variants of *S. scabiei* and shared variants among SAC species, as shown in the present work, highlights the epidemiological complexity of these mites. A high genetic diversity might provide mites with new ways of evading the host defenses (Wilson et al., 2003; Seal et al., 2021). This, combined with the vulnerability of guanaco populations, and other factors such as environmental conditions, food availability, and the infectivity of the mites themselves, could affect the general health and survival at the individual and population levels.

While the exact cause of death of the studied guanaco could not be determined, we suspect that the debilitating effects of the disease may have led to the fatal outcome (Martin et al., 2018). Although the information gathered from a single guanaco cannot be directly extrapolated to the guanaco population in Jujuy, monitoring efforts and epidemiological surveillance should be considered to obtain further information. This conclusion aligns with the findings of Montecino-Latorre et al. (2020), who emphasize the urgent need for surveillance and management plans for sarcoptic mange that may threaten biodiversity in Chile.

## 5 Conclusion

Sarcoptic mange represents a significant threat to the health and conservation of SAC. Domestic and wild camelids share *S. scabiei* strains, which underscores the need to implement health management strategies including regular monitoring and control at shared contact points to stop the transmission cycle between wild and domestic species. Future research could focus on further characterization of mite strains in the area, cross-species transmission and the risk factors associated with the dissemination of the disease, as well as on the development of treatments for domestic species and prevention protocols for wild populations of SAC.

## CRediT authorship contribution statement

**Fabiana E. Sosa:** Writing – original draft, Investigation, Conceptualization. **Melina Anello:** Writing – original draft, Investigation. **Hebe del Valle Ferreyra:** Resources. **Diego M. Medina:** Investigation. **Juan F. Micheloud:** Investigation. **Hugo M. Borsetti:** Methodology. **Nancy Hernández:** Methodology. **Mónica Florin-Christensen:** Writing – review & editing, Investigation. **Sandra R. Romero:** Supervision, Funding acquisition, Conceptualization.

## Declaration of competing interest

The authors declare no conflicts of interest.
