# Supplementary material for: Sarcoptic mange in a guanaco (Lama guanicoe) of northwestern Argentina: Clinical, histopathological and molecular studies
Source: Int J Parasitol Parasites Wildl. 2025 Mar 26;27:101062. doi: 10.1016/j.ijppaw.2025.101062 (PMC12004381; doi:10.1016/j.ijppaw.2025.101062)
Supplement: Multimedia component 1 [file mmc1.docx]

Supplementary material

Figure S1. Photograph of the guanaco at the site where it was found (Angosto de Perchel, Tilcara Department, Jujuy Province) after a warning from firefighters of the area. Note the injuries to the hind limbs caused by a dog attack.


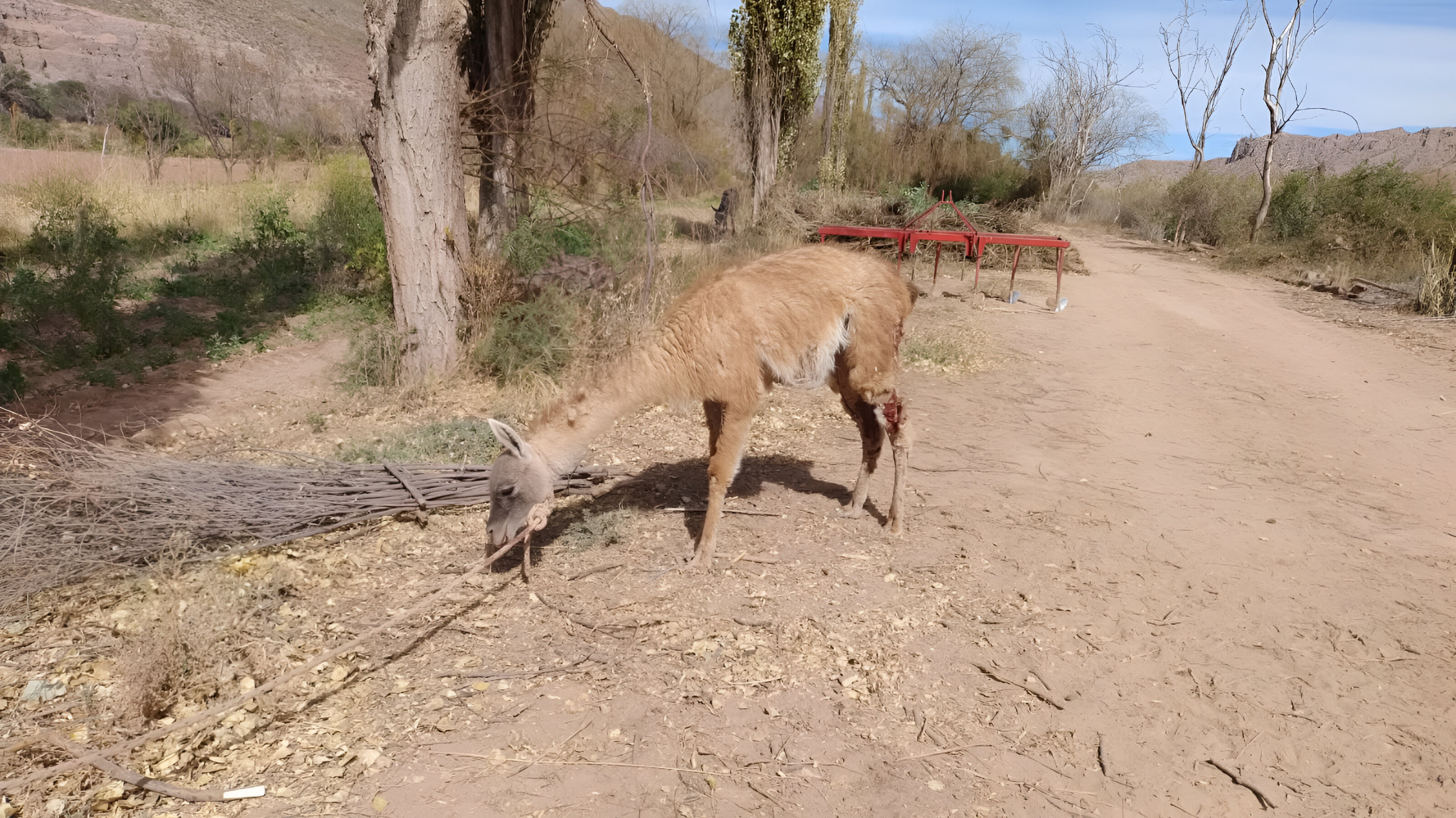


Figure S2. Nucleotide sequence alignment of *cox-1* sequences of *Sarcoptes scabiei* isolates from guanaco, llama and vicuña from Argentina, dog from China, koala from Australia, and human from Saudi Arabia.

KJ748528_dog_China GGAACTGGCTGAACTATTTATCCTCCTTTATCTAGAATCACTTATCATTCAAATATGTCT 404

PV019091_guanaco_Argentina ------GGCTGAACTATTTATCCTCCTTTATCTAGAATCACTTATCATTCAAATATGTCT 54

OL739582_vicuña_Argentina GGAACTGGCTGAACTATTTATCCTCCTTTATCTAGAATCACTTATCATTCAAATATGTCT 68

PP748549_llama_Argentina ---ACTGGCTGAACTATTTATCCTCCTTTATCTAGAATCACTTATCATTCAAATATGTCT 57

OK310847_human_Saudi_Arabia GGAACTGGCTGAACTATTTATCCTCCTTTATCTAGAATCACTTATCATTCAAATATGTCT 68

MF083743_koala_Australia GGAACTGGCTGAACTATTTATCCTCCTTTATCTAGAATCACTTATCATTCAAATATGTCT 420

******************************************************

KJ748528_dog_China GTAGATTTTACAATTGTAAGATTACATATTGCTGGAATTTCTTCTATTTTAAGTTCTATC 464

PV019091_guanaco_Argentina GTAGATTTTACAATTGTAAGATTACATATTGCTGGAATTTCTTCTATTTTAAGTTCTATC 114

OL739582_vicuña_Argentina GTAGATTTTACAATTGTAAGATTACATATTGCTGGAATTTCTTCTATTTTAAGTTCTATC 128

PP748549_llama_Argentina GTAGATTTTACAATTGTAAGATTACATATTGCTGGAATTTCTTCTATTTTAAGTTCTATC 117

OK310847_human_Saudi_Arabia GTAGATTTTACAATTGTAAGATTACATATTGCTGGAATTTCTTCTATTTTAAGTTCTATC 128

MF083743_koala_Australia GTAGATTTTACAATTGTAAGATTACATATTGCTGGAATTTCTTCTATTTTAAGTTCTATC 480

************************************************************

KJ748528_dog_China AATTTTATTGTAACTATTTATAATATAAAAATAAAAGGAATAAGATGATCAAACTTAACT 524

PV019091_guanaco_Argentina AATTTTATTGTAACTATTTATAATATAAAAATAAAAGGAATAAGATGATCAAACTTAACT 174

OL739582_vicuña_Argentina AATTTTATTGTAACTATTTATAATATAAAAATAAAAGGAATAAGATGATCAAACTTAACT 188

PP748549_llama_Argentina AATTTTATTGTAACTATTTATAATATAAAAATAAAAGGAATAAGATGATCAAACTTAACT 177

OK310847_human_Saudi_Arabia AATTTTATTGTAACTATTTATAATATAAAAATAAAAGGAATAAGATGATCAAACTTAACT 188

MF083743_koala_Australia AATTTTATTGTAACTATTTATAATATAAAAATAAAAGGAATAAGATGATCAAACTTAACT 540

************************************************************

KJ748528_dog_China CTTTTTGCTTGATCTGTTTTATTAACCTCTTTTTTATTAGTTTTCTCATTACCAGTATTA 584

PV019091_guanaco_Argentina CTTTTTGCTTGATCTGTTTTATTAACCTCTTTTTTATTAGTTTTCTCATTACCAGTATTA 234

OL739582_vicuña_Argentina CTTTTTGCTTGATCTGTTTTATTAACCTCTTTTTTATTAGTTTTCTCATTACCAGTATTA 248

PP748549_llama_Argentina CTTTTTGCTTGATCTGTTTTATTAACCTCTTTTTTATTAGTTTTCTCATTACCAGTATTA 237

OK310847_human_Saudi_Arabia CTTTTTGCTTGATCTGTTTTATTAACCTCTTTTTTATTAGTTTTCTCATTACCAGTATTA 248

MF083743_koala_Australia CTTTTTGCTTGATCTGTTTTATTAACCTCTTTTTTATTAGTTTTCTCATTACCAGTATTA 600

************************************************************

KJ748528_dog_China GCAGCAGCTTTAACAATATTATTAACAGATCGAAATTTAAGAACTTCATTTTTTGATCCT 644

PV019091_guanaco_Argentina GCAGCAGCTTTAACAATATTATTAACAGATCGAAATTTAAGAACTTCATTTTTTGATCCT 294

OL739582_vicuña_Argentina GCAGCAGCTTTAACAATATTATTAACAGATCGAAATTTAAGAACTTCATTTTTTGATCCT 308

PP748549_llama_Argentina GCAGCAGCTTTAACAATATTATTAACAGATCGAAATTTAAGAACTTCATTTTTTGATCCT 297

OK310847_human_Saudi_Arabia GCAGCAGCTTTAACAATATTATTAACAGATCGAAATTTAAGAACTTCATTTTTTGATCCT 308

MF083743_koala_Australia GCAGCAGCTTTAACAATATTATTAACAGATCGAAATTTAAGAACTTCATTTTTTGATCCT 660

************************************************************

KJ748528_dog_China ATTGGAGGAGGTGATCCTATTTTATATCAACACTTATTTTGATTTTTTGGACACCCAGAA 704

PV019091_guanaco_Argentina ATTGGAGGAGGTGATCCTATTTTATATCAACACTTATTTTGATTTTTTGGACAC------ 348

OL739582_vicuña_Argentina ATTGGAGGAGGTGATCCTATTTTATATCAACACTTATTTTGATTTTTTGGACACCCGGAA 368

PP748549_llama_Argentina ATTGGAGGAGGTGATCCTATTTTATATCAACACTTATTTTGATTTTTTGGACACCCG--- 354

OK310847_human_Saudi_Arabia ATTGGAGGAGGTGATCCTATTTTATATCAACACTTATTTTGATTTTTTGGACACCCGGAA 368

MF083743_koala_Australia ATTGGAGGAGGTGATCCTATTTTATATCAACACTTATTTTGATTTTTTGGACACCCGGAA 720

******************************************************

**Table S1.** List of samples utilized to compare the microsatellite data of the Tilcara guanaco isolates. Geographical origin, year of sampling, host species, and number of mites analyzed in each case are included.

| **Province** | **Sampling site** | **GPS** | **Sampling year** | **Host species sampled** | **Mites analyzed** |
| --- | --- | --- | --- | --- | --- |
| **San Juan and La Rioja** | San Guillermo National Park | 29°19´20,7 "S  69°22´40,1"W | 2017-2018 | *Lama guanicoe* | 11 |
|  | San Guillermo Provincial Reserve and Rodeo surroundings | 25°49´56 S  69°10´49 W | 2022-2023 | *Lama guanicoe* | 3 |
|  | Laguna Brava Provincial Reserve | 28°16’34,6´´S 68°45’27,8´´W | 2019 | *Lama guanicoe* | 5 |
|  | Laguna Brava Provincial Reserve and Famatina surroundings | 28°55'33"S 67°31'19"W | 2023 | *Lama guanicoe* | 3 |
| **Jujuy** | Coyaguayma (CY) | 22°48'32.71"S  66°22'47.81"W | 2021-2023 | *Vicugna vicugna* | 10 |
|  | Quera (QU) | 23° 3'17.13"S  65°52'35.49"W | 2019-2021 | *Vicugna vicugna* | 8 |
|  | Lagunillas del Farallón (LF) | 22°30'12.50"S  66°43'32.44"W | 2019-2021 | *Vicugna vicugna* | 15 |
|  | Lagunillas del Farallón (LF) | 22°30'12.50"S  66°43'32.44"W | 2023 | *Lama glama* | 6 |
|  | Cieneguillas (CN) | 22°02’3.18´´S  65°55’2.21´´W | 2018 | *Lama glama* | 15 |
